# Executive Dysfunction Associated With the Primary Psychopathic Features of Borderline Personality Disorder

**DOI:** 10.3389/fpsyt.2020.514905

**Published:** 2020-12-10

**Authors:** José M. López-Villatoro, Marina Diaz-Marsá, Blanca Mellor-Marsá, Irene De la Vega, José L. Carrasco

**Affiliations:** ^1^Sanitary Research Institute, Hospital Clínico San Carlos (IdISSC), Madrid, Spain; ^2^Department of Psychiatry and Medical Psychology, Faculty of Medicine, Universidad Complutense de Madrid, Madrid, Spain; ^3^Biomedical Research Networking Consortium for Mental Health (CIBERSAM), Hospital Gregorio Marañón, Madrid, Spain

**Keywords:** borderline personality disorder, psychopathy, primary factor, secondary factor, neuropsychology

## Abstract

**Purpose:** The aim of the present study is to investigate whether the presence of psychopathic features in BPD is related to dysfunction in executive functions and other neuropsychological functions in these patients.

**Methods:** 82 patients diagnosed with borderline personality disorder and 54 control subjects were studied through clinical and neuropsychological evaluation protocols and the Levenson Psychopathy Inventory.

**Results:** BPD patients showed significantly higher scores on both primary (F1) and secondary (F2) global rates of psychopathy, than controls. The results for these patients also showed a statistically significant association between high scores in primary psychopathy and deficits in executive functions. However, no associations were found between the scores of secondary psychopathy and executive dysfunction.

**Conclusion:** Primary psychopathic features present in patients with BPD are associated with patterns of executive dysfunction. It would therefore be interesting to investigate the role of cognitive rehabilitation in the empathy dysfunctions within these disorders.

## Introduction

Borderline personality disorder (BPD) is a severe and persistent mental disorder characterized by affective instability and impulsive behaviors, which affects self-image, interpersonal relationships, affectivity and behavior (1).

Several studies have found cognitive deficits in the executive functions of patients with BPD, although the results of these studies show alterations in different domains of executive function (2–5). These studies have shown significant deficits in both decision making and cognitive flexibility and planning. Some authors have related these deficits with problems in inhibitory control (6) while others find that working memory is the most affected domain, whereas the response of inhibition remains unchanged (7).

Along with the difficulties in executive function tasks, a worse performance in attention, memory and processing speed and a deficit in verbal fluency have been found in BPD patients in comparison to the control group (2, 4, 5). According to the mentioned authors, these deficits would be related to an alteration in the functioning of the prefrontal areas, which is present in BPD patients.

Psychopathy is a mental condition that negatively affects emotional processing, interpersonal relationships and self-regulation of behavior. Individuals with psychopathic characteristics are characterized by a cold and manipulative behavior in relation to others, as well as antisocial and impulsive behaviors (8).

The concept of psychopathy was first described by Koch at the end of the 19th century, as a mixture of aggressive and irresponsible behavior. This author specifically introduced the concept of “psychopathic inferiority,” which makes a difference from other forms of psychopathology, such as delusions or hallucinations or serious intellectual deficit (9).

Currently, the most accepted and replicated concept of psychopathy is Hare's two-factor model (10), from which most self-report measures of psychopathy have been built. Within psychopathy, Factor 1 (F1), or primary psychopathy, includes interpersonal and affective features of psychopathy related to the lack of guilt or remorse and the absence of fear, as well as an egocentric, manipulative attitudes and lack of empathy. Factor 2 (F2), or secondary psychopathy, includes impulsive or antisocial behaviors, such as intolerance to boredom, difficulties in planning, irresponsibility, aggression and delinquency (10).

Other scales have been developed from Hare's two-factor model, such as Levenson's Psychopathy Scale (11). This scale was validated in samples of university students (11), showing exploratory and confirmatory factor analyses with the two-factor structure. Also other authors have observed relationships between these two factors and the big five personality dimensions (12). Lynam et al. (13) considered LSRPS as a reliable and valid measurement to assess psychopathy in non-institutionalized populations.

However, despite the importance of the Hare Psychopathy Rating Scale-Revised (10), other models and measures of psychopathy have recently been developed, such as the Psychopathy Personality Inventory-Revised (14), which adds two new traits: fearless dominance and impulsive antisociality. Another scale that has been recently developed is the Tri-Arctic Psychopathy Measure (15), which evaluates three constructs of psychopathy: fearlessness, meanness, and disinhibition. In recent years, the Elemental Psychopathy Assessment (EPA) (16) has also been developed, based on the five-factor model (FFM) and which considered four defining factors of psychopathy: antagonism, narcissism, disinhibition and emotional stability.

Research focused on psychopathy factors has shown that borderline personality traits are associated more consistently with F2 traits than with F1 traits of psychopathy (17, 18). There are several studies that correlate F2 with the borderline traits, while in the case of F1 no direct correlations with the borderline features have been found, or moreover, these have been correlated in a negative way (19, 20).

Although preliminary research suggests that the relationship between psychopathic traits and BPD may be conditioned by gender, specifically by a greater presence of psychopathy in female BPD (21, 22), review of literature did not show consistent evidence for an association with sex (23).

On the other hand, the neuropsychological profile of psychopathy has been the subject of scientific controversy. Several studies (24), show a neurocognitive profile of selective attention dysfunction in the neurocognitive profile of these psychopathic subjects, which produces deficiencies in decision making. In other words, these individuals will show themselves able using information that is directly relevant to their goal, but they will show impulsive behavior and abnormal decision-making when information is beyond their immediate focus. Regarding executive functions (EF), the available evidence suggests that individuals with high levels of psychopathy do not show deficits in executive function tasks, or may even show executive functioning scores over the population mean (19).

Given these questions and considering the importance that the association between BPD, psychopathy and neuropsychological dysfunction can have for the correct diagnosis, prediction and treatment of BPD, the objective of the present study is to investigate the association between psychopathic traits and clinical characteristics of borderline personality disorder, as well as to investigate whether BPD patients with a higher rate of psychopathy show a different performance in executive functions and in attention and memory.

## Materials and Methods

### Participants

The sample studied consists of 82 patients diagnosed with borderline personality disorder, of whom 22% were men (*n* = 18), and 78% women (*n* = 64). The average age was 27.83 years (SD = 9.377). All patients had a primary diagnosis of borderline personality disorder, according to the DSM-IV-TR (1) and a moderate-severe severity (CGI, global clinical impression > 4) and a moderate-severe dysfunction (EEAG, global activity evaluation scale <65) were required to enter the study. The patients were recruited at the Personality Disorders Day Hospital of Hospital Clínico San Carlos.

Patients who presented these criteria were excluded from the study: (1) suffering from a neurological or medical disease that could affect brain functions; (2) IQ < 85; (3) having a diagnosis of schizophrenia, schizophreniform disorder or bipolar; (4) suffering a major depressive episode or a substance use disorder at the time of the study that could affect neuropsychological performance.

The sample of control participants consisted of a group of 54 people with similar characteristics (sex, age and level of studies) to the patients, of whom 11.1% were men (*n* = 6) and 88.9% women (*n* = 48). The average age was 38.02 years (SD = 9.563). The control participants were healthy -without any medical or neurological disease- and with an IQ > 85 and were recruited through advertisements in different social areas.

All patients and controls received detailed information about the study and signed written informed consent prior to their participation in the research. The clinical research study was approved by the Clinical Research Ethics Committee of the Hospital Clínico San Carlos.

### Instruments

The collection of clinical variables was performed by experienced psychiatrists and psychologists at the beginning of the study. All patients and controls were interviewed using with the Structured Interview for Personality Disorders (SCID-II) (25). Severity was measured with the Global Clinical Scale for Personality Disorders (CGI-BPD) (26) and chronicity was assessed with the Global Assessment of Functioning Scale (GAF) (27). The presence of psychopathic traits was evaluated with the Levenson Psychopathy Scale (LSRPS) (11).

The participants were evaluated with a neuropsychological battery, based on previous studies of cognitive functions in patients with BPD (28, 29). The battery consisted of several tests that evaluated three different domains: attention, memory and executive function.

#### Attention Index

This is calculated by adding standardized scores obtained from the **Symbol Digit Modality Test (SDMT)** (30) to evaluate the sustained attention and processing speed; and the standardized inverse values of the **Trail Making Test (TMT-A)** (31) which requires visual exploration, numerical ordering and visual-motor speed.

#### Memory Index

It was calculated by means of the standardized Buschke Selective Reminding Test scores (32), which measures deferred and immediate verbal memory.

#### Executive Index

This index is calculated by averaging the standardized scores of different tests: **Phonological Verbal Fluency Task (FAS)** (33), which evaluates verbal fluency; **Trail Making Test (TMT-B)** (31), which assesses cognitive flexibility; **Direct and Inverse Digits Test** (34), which measures working memory; **Stroop Test** (35), which evaluates inhibition control and **Wisconsin Card Sorting Test (WCST)** (36), which measures the capacity for abstraction, cognitive flexibility, concept development and planning.

### Statistical Analyses

Statistical analyses were carried out using the statistical package IBM SPSS Statistics (IBM Corporation, Armonk, New York, USA) version 23.0. The quantitative variables were expressed with the mean and standard deviation (SD), or median for the continuous variables that showed asymmetry. The qualitative variables were described with absolute and relative frequencies (percentages). The comparison of the quantitative variables between the two study groups was performed using the Student's *t*-test for the symmetric variables and, in the case of a non-normal distribution, the Mann-Whitney U test was applied. The linear correlation between the qualitative variables that were symmetrically distributed was evaluated by the Student's *t*-test for independent variables, establishing two samples within the BPD group and the control group based on the participants LSRPS scores. A sample would be composed by values equal to or greater than the LSRPS 75th percentile (*p* ≥ 75) and the other by values underneath that point (LSRPS *p* < 75). In every contrast performed the null hypothesis was rejected with a type I error or α error lower than 0.05.

## Results

Table 1 shows the socio-demographic variables and functioning status of patients and controls.

**Table 1:** Socio-demographic characteristics and functioning status of the BPD patients and the control group.

		**BPD group (*n* = 82)**	**Control group (*n* = 54)**
Age (mean)		27.83	38.02
Gender (percentage)	Masculine	22%	11.1%
	Femenine	78%	88.9%
Civil status	Single/Separated (%)	76%	27.8%
	Married/In a couple (%)	24%	33.4%
Current activity	Unemployed (%)	62.8%	5.6%
	Working (%)	37.2%	94.4%
Education level	Secondary studies/ Profesional studies	72.5%	85%
	Higher studies	26.3%	0%
Pharmacological treatment	Antidepressants	68.9%	0%
	Antipsychotic	44.3%	0%
	Anti-epileptic	36.1%	0%
	Benzodiazepines	59%	0%

Table 2 shows the comparison of psychopathy scores between the patients and the control group. The results show significant differences in the LSRPS scores, both in the total psychopathy index and in the primary psychopathy (F1) and secondary psychopathy (F2) indexes.

**Table 2:** Psychopathy score comparisons between the BPD patients and the control group.

	**Statistical analysis *t*; *p*-value**
LSRPS primary psychopathy	*t* = 12.186; *p* = 0.000
LSRPS secondary psychopathy	*t* = 4.164; *p* = 0.000
LSRPS total psychopathy	*t* = 16.136; *p* = 0.000

This was followed by a study on the relationship between psychopathic traits and gender, both in patients and in control subjects. The results show no significant differences in the scores of total psychopathy, primary psychopathy (F1) and secondary psychopathy (F2) of the Levenson Psychopathy Scale between men and women, both in patients (*p* = 0.058, *p* = 0.159, and *p* = 0.063, respectively) and in controls (*p* = 0.457, *p* = 0.565, and *p* = 0.144, respectively).

For the study of the relationship between the total LSRPS scores and the neuropsychological domains no linear correlations were found, so the study was conducted through two subgroups within the BPD group, one with a high score in psychopathy (*p* ≥ 75) and another with a low score (*p* < 75), which included approximately 20% of the patients at each of the upper and lower ends of the scale **scores**. The results show no differences in the neuropsychological domains of attention, memory and executive function between BPD patients with high and low psychopathy total scores (*p* = 0.570, *p* = 0.985, and *p* = 0.252, respectively).

On a second step, a more detailed study was carried out between the Levenson Psychopathy Scale components -primary and secondary psychopathy- and the neuropsychological domains. The results show no significant differences between the high primary psychopathy scores group (*p* ≥ 75) and the low primary psychopathy scores group (*p* < 75) in attention or memory (*p* = 0.096 and *p* = 0.776, respectively). However, statistically significant differences were observed in the executive function domain (Table 3), with BPD patients with high primary psychopathy scores showing a significantly lower executive performance (*p* = 0.012) than the low primary psychopathy BPD group.

**Table 3:** Neuropsychological domain differences between BPD patients with high and low LSRPS primary psychopathy scores.

	**LSRPS group**	**Mean**	**Statistical analysis *t*; *p*-value**
Attention	< *p* 75	0.0895	*t* = 0.371; *p* = 0.096
	≥ *p* 75	−0.2815	
Memory	< *p* 75	0.0114	*t* = 0.043; *p* = 0.776
	≥ *p* 75	−0.0313	
Executive function	< *p* 75	0.1078	*t* = 0.409; *p* = 0.012
	≥ *p* 75	−0.3008	

*p ≥ 75, high psychopathy scores group; p < 75, low psychopathy scores group*.

Regarding secondary psychopathy, the results showed no significant differences in the high LSRPS psychopathy scores group (*p* ≥ 75) and low LSRPS psychopathy scores group (*p* < 75) in any of the neuropsychological domains (*p* = 0.719, *p* = 0.409, and *p* = 0.387, respectively).

The study of the relationship between the neuropsychological indexes scores and the psychopathy scores was also carried out in the control group. Again, two subgroups within the control group were made based in high vs. low scores in psychopathy (*p* ≥ 75 vs. *p* < 75). Regarding the Levenson Psychopathy Scale total psychopathy index, no significant differences were found between the above subgroups regarding the attention, memory and executive function domains (*p* = 0.969, *p* = 0.297, and *p* = 0.318, respectively).

The differences according to the presence of high or low primary psychopathy (*p* ≥ 75 and *p* < 75, respectively) are illustrated in Table 4. No significant differences were found between both groups in the attention and memory indices (*p* = 0.982 and *p* = 0.438, respectively). However, there was a marked decreased trend in the executive domain in controls with greater primary psychopathy (*p* = 0.097).

**Table 4:** Neuropsychological domain differences between participants in the control group with high and low LSRPS primary psychopathy scores.

	**LSRPS group**	**Mean**	**Statistical analysis *t*; *p*-value**
Attention	< *p* 75	−0.0014	*t* = 0.006; *p* = 0.982
	≥ *p* 75	0.0044	
Memory	< *p* 75	−0.302	*t* = −0.152; *p* = 0.438
	≥ *p* 75	0.1219	
Executive function	< *p* 75	0.0815	*t* = 0.339; *p* = 0.097
	≥ *p* 75	−0.2574	

*p ≥ 75, high psychopathy scores group; p < 75, low psychopathy scores group*.

Regarding secondary psychopathy, as in patients, no significant differences were observed in the neuropsychological domains based on the participants' degree of secondary psychopathy (*p* = 0.792, *p* = 0.556, and *p* = 0.562, respectively).

## Discussion

The results of this study highlight the significant presence of psychopathic traits in borderline personality disorder compared to healthy subjects. In this sense, the findings are concordant with previous studies (18).

However, our findings do not match the previous idea that, in BPD patients, the psychopathic component is due solely to the overlapping of the borderline features with the characteristics of secondary psychopathy (F2) descried in Hare's psychopathic model (10).

In fact, in our study we also found significant differences (*p* = 0.000) between patients and controls, in the primary psychopathy index (F1), reflecting a strong positive association between the primary psychopathic traits and the BPD characteristics. This association is not in agreement with previous studies where F1 does not correlate significantly or correlates negatively with BPD features (19, 20, 37). A possible explanation for this finding relies in the potential sample differences compared to previous studies. Patients in our Day Hospital sample represent a population of subjects with severe BPD and high dysfunction, which may be related to empathic deficits and egocentric traits similar to those of primary psychopathy.

Regarding psychopathy F2, or secondary psychopathy, in our study significant differences (*p* = 0.000) were observed in the LSRPS secondary psychopathy index between the BPD group and the control group, showing a strong positive association between secondary psychopathic traits (F2) and borderline features. Some previous studies (38) have shown that patients with a borderline personality disorder and psychopathy traits have certain characteristics such as emotional instability, and aggressive and impulsive behaviors. Some (17) have gone further proposing that this is an association that could be seen as common personality patterns between the BPD and the so-called secondary psychopaths.

However, our results are not sufficient to clearly establish whether there is a real comorbidity relationship between both phenomena, BPD and psychopathy. The relationship between both phenomena is obvious, but knowing the nature of this relationship is important to determine how psychopathic traits affect the phenomenological presentation of the borderline personality disorder, and the possibility of defining different subtypes of patients with BPD according to these traits. A comorbidity relationship could assume that there are patients with BPD without psychopathy and patients with BPD with psychopathy, leading to tremendous differences in treatment and prognosis and even social and legal considerations. Conversely, if the BPD/psychopathy relationship involves more symptomatic overlap of some of the clinical aspects of BPD with those of psychopathy, it could be inferred that effective treatment of BDP would lead to improvement of the psychopathic components. Establishing the quality of these relationships could significantly improve our predictive capabilities throughout the course and outcome of treatments on our patients. This knowledge would provide greater insight into the specific treatment needs for BPD patients with high psychopathic levels.

Another important question is whether this relationship between psychopathic traits and BPD can be conditioned by gender. Although some authors consider that psychopathy and borderline disorder are actually different gender expressions of the same disorder (21, 22), the existing literature does not show consistency on this issue (23). In our results, we found no significant differences in the total scores or in the scores of the primary and secondary psychopathy components of the Levenson Psychopathy Scale between men and women in both patients and controls. Two conclusions can be derived from these results: on the one hand, there is not a greater presence of psychopathic traits according to sex and, on the other hand, it is observed that the relationship between BPD and psychopathy is similar in both sexes.

Our study also aimed to investigate whether BPD psychopathy was related to the neurocognitive deficits described in these patients. Regarding global psychopathy, including both factors, the results of our research found no significant differences between high psychopathy and low psychopathy groups when analyzing neuropsychological domain outcomes in patients and controls, suggesting that psychopathic behaviors are not due to deficiencies in neurocognitive processing.

These results are different from those observed in another study (39), which showed that subjects with a high score in psychopathy F1 had a better performance in executive functions than non-psychopathic subjects. However, the sample of the aforementioned study was composed of “successful psychopaths,” who are supposed to have relatively few characteristics of F2 in combination with strong F1 features, which would provide indirect evidence. On the contrary, in the present study patients showed a high level of dysfunction and do not correspond to the population samples included in the previous study.

For the specific case of primary psychopathy (F1) no significant differences were observed in the attention and memory indexes between the high and low psychopathy groups neither in patients nor controls. However, significant differences did appear in patients in the executive function domain, showing a negative association between the presence of F1 and executive performance. This negative association between primary psychopathy and executive functions would imply that people with high scores in psychopathy F1 have a worse performance in activities related to functions involving higher order skills as the generation, regulation, effective execution and readjustment of conducts that are addressed to objectives (40).

Finally, the presence of high secondary psychopathy in the patients group did not show any significant association with the different rates of memory, attention and executive function. These results differ from those found in previous studies (41–43), which showed a negative relationship between psychopathy F2 and executive functions. This difference may be due to the fact that previous studies used samples typified as antisocial, characterized by social impulsivity, and not by the presence of a borderline personality disorder. BPD patients in the current study show neuropsychological dysfunctions that probably reflect other aspects of the disease different from those associated exclusively with psychopathy.

Psychopathy has traditionally been linked to the diagnosis of antisocial personality disorder (APD) but recent research shows that psychopathy reflects only certain aspects of the secondary psychopathy factor (37). Antisocial disorder is usually diagnosed based on socially deviant and antisocial behavior from the age of 14 years onwards. However, there are other factors that could cause such behavior as drug addiction, certain personality traits, poverty, etc. The antisocial personality would, therefore, be only one of the factors involved, and thus, insufficient, for the diagnosis of psychopathy (8).

In this same line, Stanlenheim and Von Knorring (44) have suggested that the borderline personality disorder would be more related to psychopathy than the antisocial personality disorder itself, since the APD would be limited mainly to the alterations of the personality behavior, while the BPD includes affective and interpersonal deficits related to the primary psychopathy traits.

This study is limited by the subjective and self-evaluating nature of the Levenson psychopathic questionnaire. Future studies should contemplate the application of the Revised Hare Psychopathy Rating Scale (PCL-R) (10) as an instrument for the external evaluation of psychopathic traits. On the other hand, the influence of medication on the results of the neuropsychological tests must also be taken into account, although the effect of the covariance associated with the psychotropic drugs administration did not have a statistical significant impact in any of the tests scores.

### Conclusions

The results of the study show that in patients with BPD only the primary component of psychopathy, related to the lack of empathy and egocentricity, was related to the deficits in executive functions, while the secondary component, characterized by impulsive and behaviorally antisocial features, was not associated with neuropsychological executive dysfunctions. These findings have not been previously described and suggest that, instead of the emphasis on the study of impulsivity in BPD, it would be interesting to expand the investigation of these patients' empathic deficits and their relation to executive deficiencies, as well as the role of cognitive rehabilitation in the improvement of these traits.

## Data Availability Statement

The datasets generated for this study are available on request to the corresponding author.

## Ethics Statement

The studies involving human participants were reviewed and approved by Clinical Research Ethics Committee of the San Carlos Clinical Hospital. The patients/participants provided their written informed consent to participate in this study.

## Author Contributions

JL-V, ID, and BM-M: critical research and writing of the work. MD-M: substantial contributions to the design of the work. MD-M and JC: revision the work critically for important intellectual content. JC: final approval of the version to be published. All authors contributed to the article and approved the submitted version.

## Conflict of Interest

The authors declare that the research was conducted in the absence of any commercial or financial relationships that could be construed as a potential conflict of interest.
